# The role of plant-based dietary compounds in gut microbiota modulation in inflammatory bowel disease

**DOI:** 10.3389/fnut.2025.1606289

**Published:** 2025-05-30

**Authors:** Francis Atim Akanyibah, Chang’e He, Xiu Wang, Bo Wang, Fei Mao

**Affiliations:** ^1^Key Laboratory of Medical Science and Laboratory Medicine of Jiangsu Province, School of Medicine, Jiangsu University, Zhenjiang, Jiangsu, China; ^2^Department of Laboratory Medicine, The Affiliated People’s Hospital, Jiangsu University, Zhenjiang, Jiangsu, China; ^3^The People’s Hospital of Danyang, Affiliated Danyang Hospital of Nantong University, Zhenjiang, Jiangsu, China

**Keywords:** dysbiosis, gut microbiota, IBD, modulation, plant-based dietary compounds

## Abstract

IBD, which includes Crohn’s and ulcerative colitis, is associated with gut microbiota dysbiosis. The dysbiotic environment results in an elevation of harmful microbiota and a diminution of advantageous microbiota, leading to IBD. Interestingly, plant-based dietary compounds consisting of dietary fibers and polyphenols have demonstrated promise to be safe and successful in IBD treatment, with studies revealing that they can improve dysbiosis, increase anti-inflammatory cytokines, decrease pro-inflammatory cytokines, lower oxidative stress, and improve barrier function. Plant-based dietary compounds have shown potential to reduce IBD by regulating signaling pathways such as TGF-β/Smad, TRL-4/NF-κB/MAPK, TLR2-NF-κB, autophagy, pyroptosis, glycolysis/gluconeogenesis and amino acid metabolism, Nrf-2/HO-1, microbiota-macrophage-arginine metabolism, and bile acid metabolism. Additionally, they assist in forming short-chain fatty acids and other metabolites, which help regulate immune cells to alleviate IBD. Recent research indicates that dietary compounds, either as nanoparticles or encapsulated in nanoparticles, have shown potential in effectively treating IBD. Despite the beneficial role of plant-based dietary compounds, other studies have shown detrimental effects such as cancer promotion and exacerbation of immune responses. Therefore, this will help clinicians/individuals to plan their nutrition to prevent IBD exacerbation. This review highlights the microbiota signatures linked to IBD and examines the impact of gut dysbiosis on IBD. It also provides a comprehensive discussion of how plant-based dietary compounds can influence the modulation of dysbiotic gut microbiota in IBD. Plant-based dietary compounds hold potential for treating IBD.

## Graphical Abstract

Plant-based dietary compounds help restore a eubiotic environment in dysbiotic settings. This improves gut barrier repair and lowers inflammatory markers, easing IBD symptoms. PDC, plant-based dietary compounds; SCFA, short chain fatty acid. 
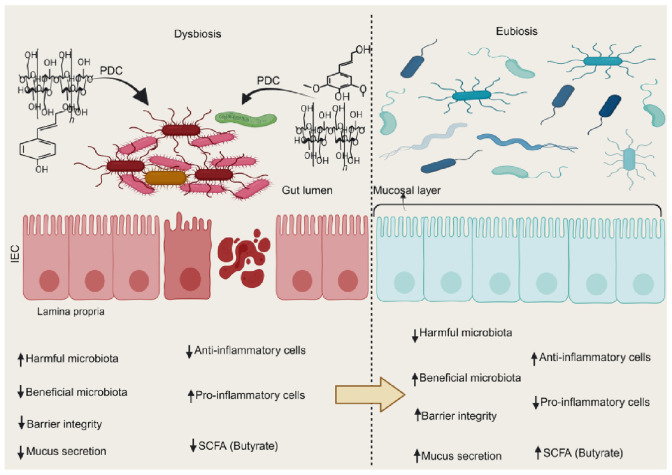

## 1 Introduction

Inflammatory bowel diseases (IBD), which include Crohn’s disease (CD) and ulcerative colitis (UC), are characterized by long-term chronic inflammation of the digestive system (1) and have a complex etiology that involves genetic susceptibility, environmental factors, and the intricate interactions between the host’s immune system and gut microbiota (2). IBD is a common condition in Europe and America, and because of changes in dietary habits, its incidence rate is rising in Asia (3). CD and UC patients often experience weight loss, diarrhea, fatigue, bloody stool, fecal urgency, mucoid stool, and abdominal pain (4).

**FIGURE 1:**
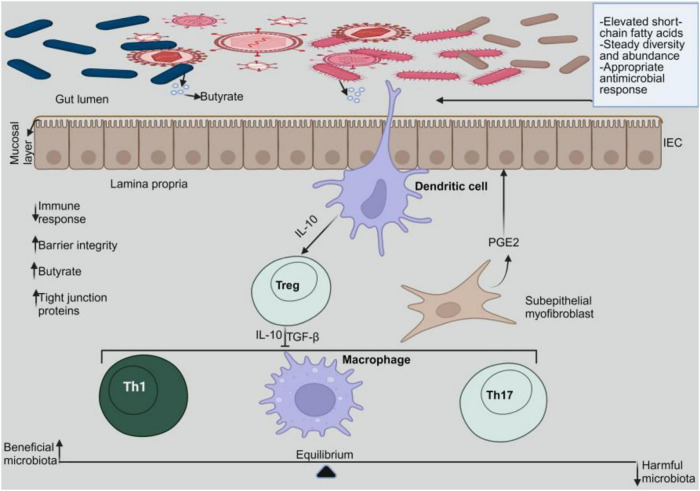
Gut microbiome homeostasis. In a homeostatic environment, the helpful microbes multiply while the toxic ones diminish. Dendritic cells interact with the beneficial microbes to emit IL-10, which stimulates Tregs. Tregs release IL-10 and TGF-β to prevent pro-inflammatory cells from activating.

The digestive tract contains numerous microbes that have evolved alongside the host immune system (5). It is now commonly known that a healthy gut flora significantly influences the host’s general health (6). The normal gut microbiota helps protect the gut mucosal barrier and control the immune system (Figure 1). It also helps break down nutrients and drugs and fight off pathogens (6). However, emerging research highlights that intestinal dysbiosis plays a critical role in both the onset and progression of IBD (7). Antibiotics, steroids, immune modulators, and 5-aminosalicylates have all been used to lessen symptoms and keep remission going in IBD (8). Nevertheless, prolonged use of these substances has been shown to cause serious toxicities, which discourage consumers (8). Thus, finding an effective treatment to restore gut microbiota to a state of eubiosis and reduce drug toxicities is essential for addressing the increasing prevalence of IBD. One potential strategy for nutritionally treating IBD with little adverse effects is the use of food bioactive substances (9).

Plant-based diets have become increasingly popular for enhancing animal welfare, improving human health, and benefiting the environment (10); consuming them offers several advantages, including improved digestive and immune system function (11). The plants’ dietary components have shown the potential to reduce colitis in mouse models by modifying the gut microbiota, lowering immune responses, and minimizing barrier damage. These compounds enhance beneficial intestinal microbes and reduce harmful ones (12–15). Diets based on plants are safe and beneficial for all phases of life, including childhood, old age, pregnancy, and breastfeeding (16). Understanding the role of gut dysbiosis in IBD is crucial for exploring additional plant-based therapies aimed at gut modulation. We therefore examine the critical function of dysbiotic gut microbiota in IBD and demonstrate how plant-based dietary compounds can successfully alter the dysbiotic gut microbiome. This will open up new paths for treating IBD symptoms.

## 2 Key microbial signatures that are involved in the dysbiosis of IBD

More than 1 trillion microorganisms live in the human body, and the gastrointestinal tract alone is home to various commensal microbes (17). The gut microbiota is an essential component of the human metaorganism that shapes physiologic host immunological responses, including host defense against infections (18). An important factor in IBD and its chronicity is the interplay among the intestinal mucosal barrier, the mucosal immune system, and a disrupted microbial makeup (19). Dysbiosis is any perturbation to the makeup or function of the microbiome (20). Microbial dysbiosis is characterized by reduced biodiversity, changed geographic distribution, abnormal gut microbiota composition, and interactions between microbiota, strains, and the host (21). IBD onset and development are associated with alterations in gut microbiota and metabolites, but the specific microbial communities affected and their potential contribution to the disease remain unclear (22, 23). Several studies have revealed gut microbiota dysbiosis, including viruses, bacteria, and fungi in IBD. The helpful microbiota decreases while the pathogenic microbiome increases. As a result, plant-based dietary components’ potential to reverse these alterations by increasing helpful microorganisms while decreasing pathogenic microbes may aid in the prevention of IBD. These will be covered in the discussion section. Table 1 summarizes the alterations in the gut microbial signatures in IBD.

**TABLE 1:** Gut microbiota signatures in IBD.

Microbiota	Model	Sequencing method	Sample	Microbial signatures	References
Bacteria	UC	16S rRNA	Stool	↓*Bacteroides* ↓*Parabacteroides* ↓*Prevotella* ↑*Actinomyces* ↑*Klebsiella* ↑*Limosilactobacillus* ↑*Streptococcus* ↑*Escherichia-Shigella*	(248)
	UC	16S rRNA, metagenomic shotgun	Stool	↓*Akkermansiaceae* ↓*Clostridiaceae* ↓*Eggerthellaceae* ↓*Lachnospiraceae* ↓*Oscillospiraceae*	(27)
	UC	16S rDNA	Stool	↑*Escherichia coli* ↑Klebsiella pneumoniae ↑Proteobacteria ↑Actinobacteria ↓Firmicutes ↓Bacteroides	(249)
	CD	16S rRNA	Stool	↓Firmicutes ↑Bacteroidetes	(7)
	CD	16S rRNA	Stool	↓Ruminococcaceae ↓Christensenellaceae ↓Erysipelotrichaceae ↓Clostridium ↓Erysipelotrichia	(250)
	CD	16S rRNA	Stool	↑*ASV 6 Escherichia/Shigella uncl*. ↑*ASV 497 Dorea uncl*., ↑*ASV-709 Subdoligranulum uncl.*	(251)
	IBD	16S rRNA	Stool	↑Coriobacteriaceae ↑Streptococcaceae ↓*Christensenellaceae* ↓*Desulfovibrionellaceae* ↓*Marinifilaceae* ↓*Rikenellaceae* ↓*Ruminococcaceae* ↓*Tannerelleaceae* ↓*Barneselliaceae*	(26)
	DSS-induced colitis	16S rRNA	Stool	↓*Lactobacillaceae* ↓*Lachnospiraceae* ↑*Bacteroidaceae*	(29)
	DSS-induced colitis	16S rRNA	Stool	↑*Firmicutes* ↑*Actinobacteria* ↓*Bacteroidetes* ↓*Verrucomicrobia*	(252)
	DSS-induced colitis	16s RNA	Stool	↓*Lactobacillus* ↓*Lachnospiraceae NK4A136* ↓*Prevotellaceae UCG-001* ↑*Bacteroides*	(253)
	DSS-induced colitis	RT-PCR	Stool	↓*Bifidobacterium* ↓*Lactobacillus* ↓*Roseburia* ↓*Akkermansia* spp ↑Prevotella spp	(254)
Fungal	CD	ITS2	Colonic mucosa samples	↑Saccharomycetes ↑Exobasidiomycetes ↑Sordariomycetes ↑*Candida glabrata* ↑*Dioszegia* ↑Cystofilobasidiaceae ↓*Leptosphaeria* ↓*Trichosporon*	(255)
	CD	ITS2 high-throughput	Stool	↑*Saccharomyces* ↑*Clonostachys* ↑*Exophiala*	(256)
	CD	ITS1-2	Stool	↑*Escherichia-Shigella* ↓*Faecalibacterium* ↓*Gemmiger* ↓*Bifidobacterium* ↓*Romboutsia* ↓*Ruminococcus* ↓*Roseburia* ↓*Fusicatenibacter*	(257)
	UC	ITS2	Stool	↑Ascomycota ↑Chytridiomycota ↑Saccharomycetaceae ↑Pleosporaceae ↑Didymellaceae ↑*Saccharomyces* ↑*Malassezia* ↑*Alternaria*	(258)
	IBD	ITS2	Stool	↑Basidiomycota- Ascomycota ratio ↓*Saccharomyces cerevisiae* ↑*Candida albicans*	(32)
	IBD	ITS	Stool	↑*Ascomycota* ↓*Basidiomycota*	(34)
Virus	UC	Deep metagenomics sequencing of VLP, 16S rRNA	Rectal biopsies	↑*Escherichia phage* ↑*Enterobacteria phage*	(36)
	UC	16S rRNA	Stool	↑Eight *Siphoviridae* VCs ↑Two Myoviridae VCs	(259)
	UC	RT-PCR and Sanger	Colonic biopsies	↑Eukaryotic *Hepadnaviridae*	(260)
	CD	Shotgun metagenomic	Stool	↑crAssphage	(37)
	CD	etagenomics and metaviromics	Stool	↑33 distinct *Torque Teno virus* species ↑*Streptomyces phage RosaAsantewaa* ↑*Escherichia phage D6/sp.* ↑*Faecalibacterium phage FP Brigit* ↑*Escherichia virus P2 4B2/4E6b*	(38)
	IBD	Shotgun metagenome	Stool	↑*Caudovirales* ↓*Petitvirales*	(261)
	VEO-IBD	Shotgun metagenome	Stool	↑*Caudovirales- Microviridae* ratio ↑*Anelloviridae*	(262)

IBD, inflammatory bowel disease; ITS, internal transcribed spacer; VEO, very early-onset; VLP, virus-like particle.

## 3 Gut microbiota dysbiosis and its impact on IBD

### 3.1 Gut microbiota diversities

Dysregulation during infancy can result in diseases later in life since the human gut flora develops and matures throughout this time. The microbiota of infants differs from that of adults in several metabolite groups, including short- and branched-chain fatty acids associated with changes in bacterial populations (24). In particular, during illness and early growth, the gut microbiota can change over time and is incredibly varied (25). Evidence from clinical (26–28) and preclinical (29–31) studies shows that the gut bacteria’s diversity is lower in people with IBD and DSS compared to controls. Reduced biodiversity of the gut fungal (32–35) and the gut virome (36–38) has also been observed in IBD both clinically and preclinically.

### 3.2 Barrier integrity disruption and influence on gut’s immunological system

**FIGURE 2:**
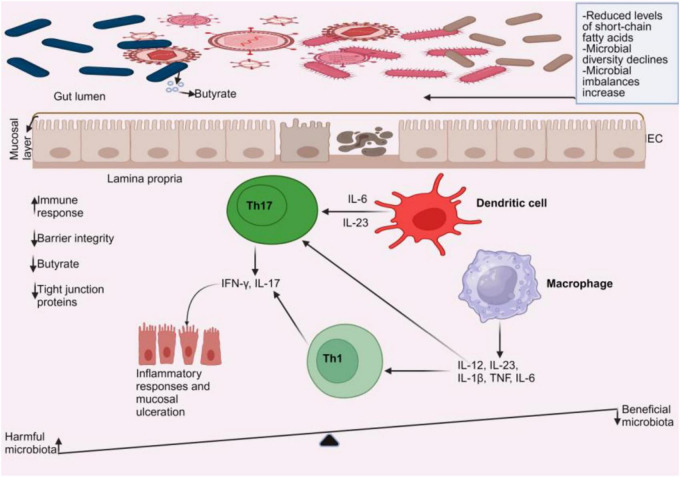
Gut microbiome dysbiosis in IBD. In a dysbiotic environment, short-chain fatty acids (such as butyrate) and gut microbial diversity decrease, accompanied by a rise in microbial imbalances. An increase in harmful microbiota leads to the activation of dendritic cells and macrophages, prompting them to produce cytokines. These cytokines stimulate T helper cells, specifically Th1 and Th17, to release more cytokines that cause mucosal ulcerations. As a result, there is a decrease in tight junction proteins and barrier integrity, along with a reduction in anti-inflammatory cells, while pro-inflammatory cells increase.

The intestinal epithelium facilitates the movement of nutrients, water, and waste products while acting as a barrier to restrict interactions between luminal contents such as the gut microbiota, the immune system, and the body (39). Tight junction proteins like Zonula occludens, occludin, and claudins are essential for maintaining the integrity of the epithelial barrier (40). Tight junctions are crucial for maintaining barrier integrity by regulating the movement of antigens through the intestinal epithelial barrier (41). Multiple studies indicate that gut microbiota comprising bacteria (42, 43), fungi (44), and viruses (45–47) can compromise intestinal barrier integrity, leading to the pathogenesis of IBD. Additionally, the gut bacteria (43, 48–50), virus (51, 52), and fungi (53–55) have shown potential to cause immunological responses in IBD. These findings imply that the gut microbiota may participate in IBD pathogenesis. Figure 2 illustrates how dysbiosis of the gut microbiome leads to compromised intestinal epithelium and heightened immune responses, exacerbating IBD. Dysbiosis increases detrimental gut microbiota, which then induces the generation of proinflammatory cytokines and disrupts the barrier function.

## 4 Plant-based dietary compounds

The American College of Lifestyle Medicine advises consuming a diet high in plant-based foods, including whole grains, legumes, nuts, seeds, fruits, and minimally processed vegetables (56). The benefits of plant-based diets for human and environmental health have made them more popular in recent years (57). Plant-based diets often provide numerous health benefits, including increased intake of essential vitamins and minerals, reduced saturated fat consumption, and increased fiber intake (56). Additionally, research has proven that plant-based diets can reduce the risk of chronic renal disease (58), improve cardiovascular health (59), combat multidrug-resistant bacteria-induced enteric disorders (60), and prevent nonalcoholic fatty liver disease (61). Polyphenols, dietary fibers, and prebiotics are the main components of plant-based diets. These are described below.

### 4.1 Types of plant-based dietary compounds

#### 4.1.1 Polyphenols

Nutritionists and food scientists are increasingly interested in the nutraceutical properties of dietary plant polyphenols, which are naturally occurring bioactive substances (62). Phenolic chemicals are significant components of plant-derived diets, as their existence correlates with health-protective properties (63). Phenolic chemicals must be liberated from the matrix during digestion in an absorbable form (bioaccessible), then absorbed and transported to the bloodstream (bioavailable) to exert their biological action (63).

##### 4.1.1.1 Sources

Plant-based foods high in polyphenols, such as fruits, vegetables, tea, coffee, wine, and chocolate, provide several health advantages (64). The gut microbiota may mediate the beneficial effects of polyphenols on host metabolism (65). Renowned for their strong antioxidant qualities, polyphenols neutralize free radicals, therefore addressing oxidative stress and helping to avoid chronic non-communicable disorders like cancer, cardiovascular problems, obesity, and diabetes (64).

##### 4.1.1.2 Types

Dietary polyphenols, such as phenolic acids, flavonoids, catechins, tannins, lignans, stilbenes, and anthocyanidins, are prevalent in grains, cereals, legumes, vegetables, spices, fruits, chocolates, and drinks including fruit juices, tea, coffee, and wine (66). Additionally, curcuminoids are phenolic chemicals frequently added to food as a spice, color, and additive. They serve as a medicinal agent (67).

###### 4.1.1.2.1 Phenolic acids

One important type of dietary polyphenols that are naturally occurring antioxidants is phenolic acids (68). Plant metabolites called phenolic acids are present in various parts of the plant kingdom (69). Mohammed and his team used phytochemical analysis to find phenolic acids in *Ephedra alata* Decne. These acids include p-coumaric acid, ferulic acid, ellagic acid, caffeic acid, vanillic acid, rosmarinic acid, and chlorogenic acid (70). Researchers have demonstrated that phenolic acids slow down the progression of osteoarthritis by reducing the expression of catabolic factors, mitigate alcohol-induced liver disease by altering the hepatic circadian rhythm signaling pathway through the gut microbiota-NPAS2 axis, prevent inflammation and ferroptosis by controlling the AMPK/mTOR/HIF-1 signaling pathway, alleviate *S. aureus*-induced endometritis in mice, shield testicular injuries caused by cyclophosphamide, and reduce vascular endothelial growth factor-induced angiogenesis and endothelial permeability. Phenolic acids have also been found to effectively treat neurotoxicity brought on by exposure to neuroendocrine disruptors, reduce splenic tissue inflammation, balance oxidative stress in carp via the nuclear factor erythroid 2-related factor 2 (Nrf2)/NQO-1 pathway, and enhance spleen apoptosis (71–77).

###### 4.1.1.2.2 Flavonoids

Flavonoids are substances that are found in nature and have a variety of health benefits (78). Numerous plants, fruits, vegetables, and leaves contain phytochemicals called flavonoids, which may have uses in medical chemistry (79). Proanthocyanidins are byproducts of flavonoid biosynthesis, consisting of oligo- or polymers made from monomeric flavan-3-ols (80). The degree of unsaturation and oxidation of the C ring and the carbon to which the B ring is linked divide flavonoids into subgroups. Isoflavones are flavonoids with a B ring connected to a C ring at position 3, and neoflavonoids have a B ring connected at position 4. Flavonoids with a B ring connected at position 2 can be further classified into subgroups based on the C ring’s structural characteristics. These subcategories include anthocyanins, chalcones, flavones, flavonols, flavanones, flavanonols, and flavanols or catechins (81). Researchers have proven several advantages from these substances. For instance, increased anthocyanin intake has been associated with a decrease in cardiovascular disease mortality (82). Chalcone T4 also inhibits inflammation in periodontal tissues and the loss of alveolar bone (83). Isoorientin (a natural flavone) therapy in mice with excisional wounds improved tissue healing (84). There is evidence that the natural flavonol kaempferol and flavanonol dietary dihydromyricetin can help treat rheumatoid arthritis and protect against growth retardation and intestinal damage caused by enterotoxigenic *Escherichia coli*, respectively (85, 86). Other types of flavonoids have also shown promise in slowing the progression of nonalcoholic fatty liver disease, lowering signs of neuroinflammation and cell death in the hippocampus in people with Gulf War Illness, and possibly being used as a treatment for the Omicron version of SARS-CoV-2 (61, 87, 88).

###### 4.1.1.2.3 Curcuminoids

Curcuminoids, commonly used as pigment spices, are phenolic chemicals with antiviral, antitumor, anti-HIV, anti-inflammatory, antiparasitic, anticancer, and antifungal properties. The main active and consistently bioactive components are curcumin, bisdemethoxycurcumin, and demethoxycurcumin (89). Curcumin’s strong anti-inflammatory qualities and regulatory impact on the gut flora make it a research hotspot for IBD treatment (90). Also, bisdemethoxycurcumin holds enormous promise for creating powerful inhibitors that reduce the likelihood of deadly amyotrophic lateral sclerosis (91). Demethoxycurcumin has been shown to upregulate peroxisome proliferator activated receptor γ (PPARγ), which inhibits the growth of cervical cancer cells (92).

###### 4.1.1.2.4 Tannins, lignans, stilbenes

These chemicals, recognized as dietary polyphenols from various sources, exhibit beneficial effects in disease management. For instance, Pterostilbene, a naturally occurring stilbene, has anticancer properties in head and neck cancer cells and prevents liver damage from alcohol consumption, both acute and chronic (93, 94). *Schisandra chinensis* (Turcz.) Baill’s lignan-enriched extract offers protection against Parkinson’s disease (95). After mandibular molar extraction, green tea tannin has been shown to stop bleeding much better than aqueous and methanolic extracts (96).

#### 4.1.2 Dietary fibers

Fiber consumption improves metabolic homeostasis in both humans and rodents, which in turn leads to changes in the gut microbiota (97). Due to the absence of the digestive enzyme necessary for fiber digestion, dietary fiber is a nondigestible form of carbohydrates in humans (98). Dietary fibers’ structure dictates the metabolic variations and alterations in the gut microbiota brought about by fermentation, which in turn influence the health impacts of gut microbes (99). Consuming dietary fiber, especially insoluble fiber from fruits, vegetables, and other foods, may lower the risk of breast cancer, particularly in premenopausal women (100). Zheng et al. (101) found that total, insoluble, or soluble dietary fibers taken from highland barley can help mice on a high-fat diet (HFD) lose weight, change their blood lipid profiles, and heal damaged tissues (101).

##### 4.1.2.1 Classification of dietary fibers

Dietary fiber falls into the soluble or insoluble category based on its water solubility characteristics (98). Soluble fibers comprise pectin, inulin, resistant starch, β-glucan, gums, and mucilages (102–104), while insoluble fibers comprise cellulose, hemicellulose, and lignin (103, 104).

###### 4.1.2.1.1 Soluble fibers

####### 4.1.2.1.1.1 Pectin

Apple and citrus peels are currently the main ingredients used in commercial pectin manufacturing (105). Pectin is a collection of intricate polysaccharides naturally occurring in diverse plants and linked to numerous advantageous health benefits (106). Pectins are dietary fibers recognized for their various positive immunomodulatory effects and their role in managing and preventing different inflammatory disorders (107, 108). Pectins can be classified as high methoxyl pectin or low methoxyl pectin based on the degree of esterification (107). A study has demonstrated that pectin prevents ileitis (109). Liu and colleagues found that pectin from comfrey roots could lessen colon damage from DSS in rats and repair the intestinal barrier (110). In a pilot study, supplementing healthy volunteers with citrus low-methoxy pectin lowers inflammation and anxiety levels (111).

####### 4.1.2.1.1.2 Inulin

As a reserve polysaccharide, inulin, a soluble dietary fiber, is present in over 36,000 plant species (112). Jerusalem artichokes, chicory, onions, garlic, barley, and dahlia are the main sources of inulin (112, 113). The food industry commonly uses chicory roots and Jerusalem artichoke tubers as raw materials for inulin manufacturing (112). A well-known prebiotic component that has been shown to alter the gut flora and its metabolic processes is inulin (114). This suggests that inulin may possess prebiotic properties. Researchers have demonstrated that inulin prevents atherosclerosis by boosting the intestinal barrier and gut microbiota, decreasing inflammation, and increasing lipid metabolism (115). Research suggests that inulin may have antidiabetic effects by enhancing insulin resistance and insulitis, and reducing obesity progression by modulating gene expression in the prefrontal cortex via endocannabinoids (116, 117).

Small interfering RNA (siRNA)-mediated therapy has shown potential in treating various illnesses by inhibiting gene expression, including those involved in cancer initiation and spread, making it a promising treatment option (118). Interestingly, researchers have applied nanoparticles to inulin to treat diseases. For instance, researchers developed systemically administered nanoparticles using inulin modified with α-cyclam-p-toluic acid (CPTA) (IC) and siRNA against p53, which preferentially concentrate in damaged kidneys and significantly decrease p53 expression (119). Mice with cisplatin-induced acute kidney injury showed improved renal function overall and decreased tubular cell death, renal injury, and inflammation due to selective p53 knockdown (119).

####### 4.1.2.1.1.3 β-glucan

β-glucans are a class of β-D-glucose polysaccharides (glucans) found naturally in grain, fungal, and bacterial cell walls (120). A non-starch-soluble polysaccharide, β-glucan, is found in many foods, including barley, oats, yeast, mushrooms, bacteria, and algae (121). Observations show that the structural features of β-glucan, such as specific glycosidic connections, monosaccharide compositions, molecular weight, and chain conformation, influence its physiochemical and biological properties (121). Enzymes called β-glucanases break down β-glucan into glucose and cello-oligosaccharides (120). β-glucans have demonstrated the ability to elevate reactive oxygen species and induce apoptosis in melanoma cells, alter local innate responses in ewes by preserving the integrity of the mammary epithelial barrier, and exhibit unique immune-modulating properties, rendering them compelling adjuvants for prospective allergy therapies (122–124).

####### 4.1.2.1.1.4 Gums

Gums are carbohydrate-based biomolecules that bind water and form gels. Types include exudate, mucilage, and seed gums. Plant gums, due to their bioavailability, are significant and have been used by humans since prehistoric times for various purposes (125). Plant-derived gums and mucilages are suitable pharmaceutical excipients due to their non-toxicity, stability, availability, regulatory compliance, cost-effectiveness, and adaptability to specific requirements (126). According to a phytochemical analysis, *Prunus armeniaca* and *Prunus domestica* gum include proteins, carbohydrates, and saponins (127). These gums exhibit potential in pharmaceutical compositions (127, 128). Furthermore, gum can serve as a medium for the oral administration of protein pharmaceuticals (129). Hydrogel made of chicha gum has demonstrated promise as a wound-healing agent (130).

####### 4.1.2.1.1.5 Mucilages

The food industry is interested in mucilage, a hydrophilic biopolymeric substance in high concentrations in agricultural by-products such as the peel of cactus fruits, due to its high dietary fiber content, antioxidant activity, and gelling and thickening properties (131). Plant parts like seeds, rhizomes, and roots can yield mucilage (132). In nature, it is a polysaccharide made up of large sugar molecules and uronic acid components (132). Okra’s mucilage and flesh may be potential remedies for preventing metabolic dysfunction (133). In mice with alloxan-induced diabetes, *Abelmoschus esculentus* mucilage has good antioxidant potential and hypoglycemic and hypolipidemic effects (134).

####### 4.1.2.1.1.6 Resistant starch

Humans have long used resistant starch (RS) as a food source, and almost all starchy foods contain it (135). RS is a type of starch that can pass through the small intestine and reach the colon, despite being indigestible by human pancreatic amylase (135). Five different RS types modulate gut microbiota to respond differently to chronic illness (136). Short-chain fatty acids (SCFAs) bridge the gut microbiota and RS, and RS has the potential to improve the metabolism of the gut microbiota and increase the population of beneficial bacteria in the gut (136). RS may enhance body weight and carbohydrate and lipid metabolism (137). The RS diet has shown positive effects on renal function indicators and uremic toxin levels in patients with chronic kidney disease (138).

####### 4.1.2.1.1.7 Prebiotics

Prebiotics are indigestible food fibers that have undergone selective fermentation. They specifically encourage the growth of one or more bacterial genera in the gut, which benefits the host’s health (139). Prebiotics that are helpful to human health fall into two major categories: galactooligosaccharides and fructooligosaccharides (140). Prebiotics can feed the gut microbiota, and their breakdown releases SCFAs into the bloodstream that affect the gut and other external organs (140). Consuming prebiotics such as arabinoxylan oligosaccharides and inulin-type fructans can boost the quantity of bifidobacteria in the colon (141). Bifidobacteria play various roles, including breaking down indigestible carbohydrates, protecting against infections, synthesizing vitamin B, antioxidants, and conjugated linoleic acids, and activating the immune system (141). Supplementing male rats with oligofructose prebiotic fiber has been shown to lessen the effects of a diet heavy in fat and sugar and to prevent knee joint deterioration (142). Notably, the majority of prebiotics are soluble fibers.

###### 4.1.2.1.2 Insoluble fibers

####### 4.1.2.1.2.1 Cellulose

Cellulose is the most prevalent polysaccharide on Earth. It can be found in a wide variety of places, including the cell walls of plants and wood, certain types of bacteria, algae, and tunicates—the only known animals that possess cellulose (143). Cellulose can be categorized into fiber, microfibril/nanofibril, or micro/nanocrystalline cellulose based on the selection of physical characteristics, sizes, and shapes (143). SCFAs produced by cellulolytic bacteria from dietary fiber are vital for maintaining gut health and optimizing fiber use (144). The gut microbiota is essential for digesting cellulose, the primary component of plant fiber, in humans and all other mammals. The human gut microbiota contains ruminococcal species that form multi-enzymatic cellulosome structures, which are functional and capable of breaking down plant cell wall polysaccharides. A species closely related to humans likely originated in ruminants’ guts and spread to humans during domestication. It acquired genes from other gut microbes and underwent diversification and diet-related adaptation. These species are common among hunter-gatherers, ancient people, and rural groups but are uncommon in populations from industrialized countries, suggesting they may go extinct due to Westernized lifestyles (145). Microparticles made of highly crystalline seaweed cellulose may help control gut microbial dysbiosis and reduce obesity and metabolic syndromes linked to a high-fat, high-sugar diet in mice (146). Also, cellulose nanocrystals have demonstrated the potential to enhance intestinal retention and assist in body weight management (147).

####### 4.1.2.1.2.2 Hemicellulose

Plant cell walls contain polysaccharides called hemicelluloses, which have beta-(1– > 4)-linked backbones (148). Around one-third of wall biomass comprises hemicelluloses, including heteromannans, xyloglucan, heteroxylans, and mixed-linkage glucan (149). Research suggests finger millet-derived arabinoxylan and *Delonix regia* galactomannan can be used as nutraceuticals to control high-fat diet-induced obesity and enhance wound healing in murine cutaneous wounds by increasing transforming growth factor-beta (TGF-β) levels (150, 151). Lemieszek and colleagues have also demonstrated *Cantharellus cibarius* branched mannans as a novel treatment option for colon cancer (152).

####### 4.1.2.1.2.3 Lignin

Lignin, a dietary fiber derived from plant cell walls, has several biological anti-inflammatory and antioxidant properties (153). Lignin is a complex polymer of phenylpropane units that exhibits extensive cross-linking (154). Common lignin structures found in the insoluble dietary fiber of fruits and vegetables comprise glycerols, spirodienones, dibenzodioxocins, and lignin precursors in the gut known as resinols (155). The nature of the cell wall, particularly the amount of lignin present, significantly influences the gut microbiota and fermentation results (156). Lignin prevents ferroptosis in UC by interacting with GPR37 and triggering the extracellular signal-regulated kinase-Nrf2-glutathione peroxidase 4 (GPX4) signaling pathway, providing new clinical intervention concepts for UC treatment (153). Additionally, lignin-carbohydrate complexes reduce the neurotoxicity of bisphenol A in zebrafish by reducing oxidative stress (157).

## 5 Role of plant-based dietary compounds in modulating the gut microbiome to repair IBD

### 5.1 Dietary fibers

#### 5.1.1 Soluble dietary fibers

**FIGURE 3:**
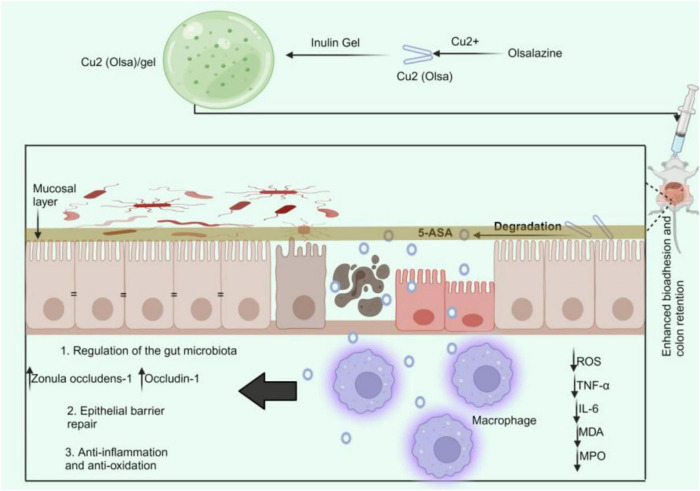
Cu2(Olsa)/Gel treatment of IBD. The oral delivery of Cu2(Olsa)/Gel improves bio-adhesion and colon retention. This makes it easier for 5-ASA to penetrate the inflammatory tissue slowly. It lowers proinflammatory cytokines and speeds up the repair of the epithelial barrier by fighting free radicals and inflammation. This leads to colitis alleviation. 5-ASA, 5-aminosalicylic acid; IL-interleukin; MDA, malondialdehyde; MPO; myeloperoxidase; Olsa, olsalazine; ROS, reactive oxygen species; TNF-α, tumor necrosis factor-alpha.

Numerous studies have demonstrated that inulin can avert various disorders, including IBD. Researchers have recognized additional inulins for their prebiotic characteristics. Inulin enhances beneficial microbiota and diminishes detrimental microbiota. These strategies mitigate inflammation and preserve the integrity of the gut mucosal barrier. For instance, *Lactobacillus rhamnosus* 1.0320 combined with inulin can alleviate colitis caused by DSS, reduce the disease activity index score of colon tissue damage, and increase IL-10 expression while downregulating IL-1β, IL-6, TNF-α, and myeloperoxidase (MPO) (158). Additionally, the combination dramatically increases the abundance of *Bacteroidales S24-7* in enteritis-affected mice while decreasing the abundance of *Lachnospiraceae* and *Ruminococcaceae* (158). Cao and team also found that the inulin gel matrix can prolong the residence time of polypyrrole (PPy) nanozymes and pirfenidone (PFD) in the gastrointestinal tract, reducing pro-inflammatory cytokine levels, repairing the intestinal epithelial barrier, and suppressing intestinal fibrosis through sustained reactive oxygen and nitrogen species scavenging and attenuation of the TGF-β/Smad signaling pathway. The gut microbiota was altered, enhancing the presence of beneficial genera like *Coprococcus* and *Oscillospira*, which aid in butyrate production, a crucial fatty acid for intestinal barrier restoration (159). Zhang and his team also found that in three animal models of IBD, the Cu2(Olsa) nanoneedle-inulin gel composite significantly reduced colitis by promoting the repair of the epithelial barrier through anti-inflammatory and antioxidant therapies while downregulating levels of pro-inflammatory cytokines (Figure 3). The inulin gel composite containing Cu2(Olsa) nanoneedles reduced the number of harmful microorganisms, including *Proteobacteria* (160). Various studies have demonstrated the potential of inulin in alleviating different ailments. For instance, inulin may improve metabolic diseases by altering the gut microbiota and enhancing the generation of SCFAs, potentially mediated by the angiopoietin-like protein 4-related signaling pathway. Dietary inulin improved gut microbiota dysbiosis, reduced the loss of *Bacteroidetes*, inhibited the growth of *Firmicutes*, and enhanced the ratio of *Firmicutes* to *Bacteroidetes* (161). Also, inulin improves anxiety- and depression-like behaviors in alcohol-dependent withdrawal mice by increasing the number of *Faecalibacterium* and *Roseburia*, boosting the generation of SCFAs, and regulating serotonin metabolism (162). Additionally, inulin27 has significantly decreased rats’ systemic glucose levels and weight gain from an HFD. Inulin7 reduced levels of *Lachnospiraceae* linked to metabolic disorders while promoting beneficial *Bifidobacteriaceae* taxa (163). This indicates that inulin enhances gut microbiota in several disorders, including IBD. However, researchers have found that inulin may induce carcinogenesis in IBD. Tian and team found that following DSS treatment, mice fed inulin showed significant colitis, while mice who completed the research showed substantial colon cancer. Inulin caused a shift in gut flora, which supported the increase of succinate in the gut lumen. Interestingly, inulin-fed mice showed a rise in Bacteroidota cecal abundance (164). In a different study, Hoving and the team found that although inulin has prebiotic effects, it did not reduce hypercholesterolemia or atherosclerosis in *E3L.CETP* mice. However, it did lead to hepatic inflammation when combined with a high cholesterol intake. Inulin significantly increased the abundance of *Coprococcus* and *Allobaculum* in mice, while decreasing the abundance of *Bacteroides*, *Parabacteroides*, *Prevotella*, *Micispirillum*, *Clostridium*, and *Coprobacillus* compared to control mice (165). These findings indicate that inulin may exhibit diverse roles in several disorders, including IBD.

As previously mentioned, the two primary prebiotics are galactooligosaccharides and fructooligosaccharides. Therefore, Koleva and her team discovered that fructo-oligosaccharides markedly alleviated colitis in rats. While FOS increased the number of *Bifidobacterium* spp., both fructans (FOS and inulin) decreased the expression of the genes for Clostridium difficile toxin and Clostridium cluster XI, which was associated with a decrease in chronic intestinal inflammation (166). Another study found that FOS lowers colitis and levels of the pro-inflammatory cytokine IL-1β in rats given rat chow. FOS increased the number of copies of Bifidobacteria, *Enterobacteriaceae*, and butyryl-CoA transferase genes while decreasing those of *Clostridium* cluster IV. In rats given FOS, the relative content of acetate was noticeably higher (167). In a different study, treatment with fructooligosaccharides improved the changes in pathology in transgenic mice and cognitive impairments. Fructooligosaccharides therapy enhanced the abundance of *Lactobacillus* while decreasing the abundance of *Helicobacter* in the transgenic group (168). Conversely, in mice with stress-induced IBS, the injection of FOS increases gut inflammation and visceral hypersensitivity (169). Another study also found that chitooligosaccharides (COS) and *Clostridium butyricum* decreased clinical symptoms, enhanced colonic morphology, controlled cytokine levels linked to inflammation, prevented the activation of the TLR-4/NF-κB/MAPK signaling pathway, preserved intestinal barrier function, and increased intestinal homeostasis by adjusting the diversity and composition of the gut microbiota (170). These findings indicate that prebiotics may influence the gut microbiota in several disorders, including IBD.

The gums, categorized as soluble dietary fibers, modulate the gut microbiota to prevent IBD. Partially hydrolyzed guar gum (PHGG) lowers increases in myeloperoxidase activity, TNF-α protein, and mRNA expression in the colonic mucosa and repairs damage in the colon caused by TNBS. Mice treated with PHGG exhibited markedly higher caecal proliferation of the *Bacteroides fragilis (B. fragilis)* group, the *Clostridium leptum* subgroup (Clostridium cluster IV), and the *Clostridium coccoides* group (*Clostridium* cluster XIVa), along with increased SCFAs such as propionic acid and butyric acid (171). The study focused on gut microbiota, excluding significant bacteria like *Bifidobacteria*, and assessed microbiota and SCFA after TNBS-induced colitis, suggesting further research is needed to address these issues (171). Nevertheless, a study by Paudel and team found that following DSS intervention, mice given a guar gum-containing diet showed more severe colitis than the control group. Primarily, guar gum enhances Actinobacteriota, particularly *Bifidobacterium*. In the guar gum diet-fed mice, this change in the makeup of the gut microbiota promoted the luminal accumulation of intermediary metabolites lactate and succinate (172). These changes may result from the type of gum diet (refined and partially hydrolyzed guar gum). In a different study, PHGG partially inhibited the development of non-alcoholic fatty liver disease in mice through the gut-liver axis by modifying the microbiota and the resulting SCFA profiles. PHGG dramatically raised the prevalence of *Clostridium* subcluster XIVa and *Bacteroides* in the cecum. Furthermore, PHGG therapy significantly raised SCFA levels in the cecal samples, specifically butyric acid, acetic acid, propionic acid, and formic acid (173). Therefore, PHGG may similarly prevent IBD via regulating the gut microbiota and producing SCFAs.

**FIGURE 4:**
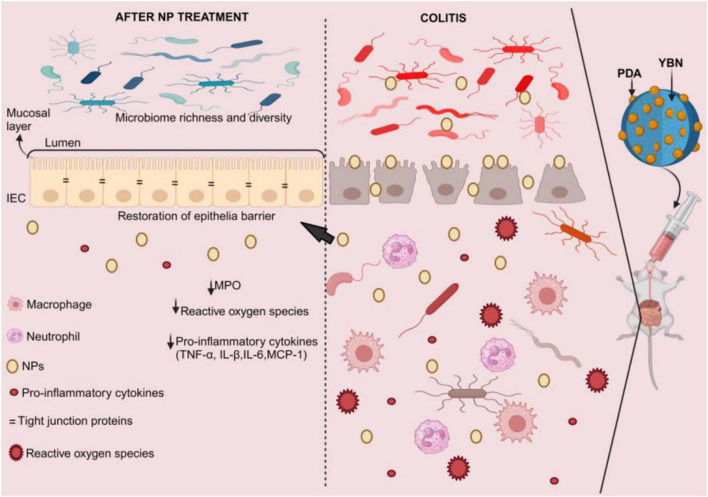
YBNs@PDA intervention in IBD. Due to the mucoadhesive PDA layer that enables YBNs to adhere to inflammatory colon mucosa, these nano-agents extended the time of retention in the intestine, allowing them to optimize the bioavailability of the medicine. This coating also enhances the core YBNs’ potential for beneficial gut flora regulation, enhancing their effectiveness in treating inflammatory colon conditions. This restores the epithelial barrier, lowers the levels of reactive oxygen species and pro-inflammatory cytokines, and raises the diversity and richness of the gut microbiota. MPO, myeloperoxidase; NPs, nanoparticles; PDA, polydopamine; YBNs, yeast β-glucan nanocomplex.

As previously mentioned, β-glucans, which are soluble dietary fibers, have shown the ability to reduce IBD by influencing gut flora. Additionally, certain β-glucans can be encapsulated with nanoparticles to alleviate IBD. For instance, in an acute colitis mouse model, oral administration of a yeast β-glucan nanocomplex coated with bio-adhesive polydopamine (YBNs@PDA) has been demonstrated to improve therapeutic efficacy while restoring epithelial barriers, lowering ROS levels, and minimizing systemic drug exposure (Figure 4). YBNs@PDA significantly increased the number of *Bifidobacterium* and *Lachnospiraceae NK4A136*, two probiotics that are essential for reducing colitis by maintaining gut homeostasis (174). In a different study, oat β-glucan (ObG) supplementation changed the gut microbiota profile, enhancing the generation of butyrate in the intestines of both 4-week-old pups and their dams, and increased spatial memory and cognition at week 8 (pups). The Firmicutes phylum was significantly more prevalent in the ObG group’s dams and pups than in the control group (175).

The treatment of resistant starch from purple sweet potato (PSPRS) significantly improved colon inflammation and pathological characteristics in a dose-dependent manner compared to DSS-induced colitis in mice. The PSPRS therapy group had significantly higher levels of putative probiotic bacteria, including *Lactobacillus*, *Alloprevotella*, *Lachnospiraceae*_NK4A136_group, and *Bifidobacterium*, as well as a higher ratio of *Firmicutes* to *Bacteroidetes* (176). High PSPRS dosages, meanwhile, markedly raised butyrate, propionate, and acetate production (176). Nonetheless, the relationship between microbiota and PSPRS structure is unclear, necessitating further research. Furthermore, in DSS-induced colitis mice, a single dose of PSPRS was applied to the gut microbiota and SCFAs; additional doses ought to be selected in subsequent studies to elucidate PSPRS’s anti-inflammatory action (176). The type of RS significantly impacts the gut microbiota’s fermentation of SCFAs, with different RS types possessing unique structural traits that ultimately lead to SCFA generation (177).

Li et al. (178) found that the reduction of *Salmonella* abundance and the inactivation of TLR2-NF-κB signaling may explain why dietary pectin improved tight junctions, oxidative stress, and colitis caused by *Salmonella typhimurium*. Interestingly, the study also discovered that *Salmonella typhimurium* markedly increased the colon’s p-NF-κB/NF-κB ratio and TLR2 protein expressions (178). These findings imply that dietary pectin may ameliorate *Salmonella typhimurium*-induced colitis by inhibiting the TLR2/NF-κB signaling pathway and oxidative stress.

#### 5.1.2 Insoluble dietary fiber

Insoluble dietary fibers, including cellulose and xylans, have shown promise in modulating gut microbiota to help prevent IBD. In this case, Kim and colleagues discovered that high-cellulose diets (HCD) protect mice from DSS-induced colitis, while low-cellulose diets (LCD) increase intestinal inflammation. Compared to mice fed LCD, mice fed HCD had a higher relative abundance of the genus *Akkermansia*. *Akkermansia muciniphila*, given orally to LCD-fed mice, improved colitis, lengthened crypts, and expanded goblet cells. Dietary cellulose reduces inflammation in the gut by regulating gut microbiota and lipid metabolism (13). Additionally, mice fed HCD had greater levels of 13(S)-HODE (hydroxyoctadecadienoic acid), while the precursor of 9(S)-HODE (hydroxyoctadecadienoic acid) was higher in LCD-fed mice than in HCD-fed mice (13). Moreover, cellulose, when used at an optimal dosage of 1.5 g/kg, has been proven to reverse the pathological process of colitis by preventing colon damage, balancing oxidative stress, controlling inflammation, and preventing weight loss. Cellulose primarily targets and regulates the number of *unclassified Lactobacilli*, *Bacteroides*, *Faecalibaculum*, and *norank Lactobacilli.* Additionally, cellulose raised the levels of SCFAs, including total SCFAs, butyric acid, propionic acid, and valeric acid (179). In another study, treating citric acid-crosslinked carboxymethyl cellulose nanofibers shields mice against diet-induced obesity and metabolic dysfunction by increasing energy expenditure, decreasing food intake, and enriching probiotics like *Bifidobacterium* (180).

Zhao et al. (181) found that ferulic acid-derived lignin nanoparticles (FALNP) significantly reduced pathogenic symptoms by controlling the gut microbiota and lowering oxidative stress in a mouse model of acute colitis. The mice treated with FALNP showed a considerable rise in *Lactobacillus* and Lachnospiraceae_NK4A136_group. Through intestinal microenvironment regulation, FALNP can tolerate the gastric acid environment and significantly alleviate pathological symptoms in colitic mice (181). The FALNP-based delivery system, despite its versatility, is not selective for inflammatory areas, necessitating further biological or chemical modification for improved oral delivery therapy of intestinal illnesses (181).

Xylan butyrate ester (XylB) treatments can balance pro- and anti-inflammatory cytokines, lessen damage to mice’s guts, and restore gut microbiota that was harmed by DSS treatment. This lowers the number of *Oscillibacter*, *Ruminococcaceae UCG-009*, *Erysipelatoclostridium*, and *Defluviitaleaceae UCG-01* genera. XylB increased colon butyrate concentration, decreased histone deacetylase (HDAC) activity, increased G-protein-coupled receptor 109A protein expression, and activated autophagy and NF-κB, resulting in anti-inflammatory effects (182). In a different study, supplementing with specific xylans, such as arabinoxylan derived from rice bran (RAX), dramatically reduces obesity from a high-fat diet. RAX decreases the relative abundance of pro-inflammatory bacteria such as *Anaerotruncus*, *Helicobacter*, *Coprococcus*, and *Desulfovibrio* while increasing the relative abundance of anti-inflammatory bacteria like *Bifidobacterium and Akkermansia* (183). Table 2 summarizes the role of dietary fibers in regulating the gut microbiota to prevent IBD.

**TABLE 2:** Dietary fibers that regulate the gut microbiota signatures to prevent colitis.

Dietary fiber	Model	Sequencing method	Sample	Mechanism of action	Impact on gut microbiota signatures	Outcome	Reference
**Soluble**							
**Inulin**							
*Lactobacillus rhamnosus* 1.0320 + inulin	DSS-induced colitis	16S rDNA high-throughput	Stool	Augments anti-inflammatory cytokines, diminishes pro-inflammatory cytokines, and alters gut microbiota.	↑*Bacteroidales S24-7* ↓*Lachnospiraceae* ↓*Ruminococcaceae*	Relieves DSS-induced IBD.	(158)
Ternary PPy/PFD@Inulin gel^&^	DSS-induced UC	16S rRNA	Stool	Decreases pro-inflammatory cytokines, enhances gut barrier repair, modulates gut microbiota, and inhibits the TGF-β/Smad signaling pathway.	↑*Coprococcus* ↑*Oscillospira*	Reduces IBD and intestinal fibrosis.	(159)
Cu2(Olsa) nanoneedle-inulin gel composite^&^	DSS-induced UC	16S rRNA	Stool	Downregulates pro-inflammatory cytokine concentrations and facilitates epithelial barrier restoration via anti-inflammatory and antioxidant interventions	↓*Proteobacteria*	Decreases colitis	(160)
**Gums**							
PHGG	TNBS-induced colitis	16S rRNA	Stool	Inhibits the onset of TNBS-induced colitis in mice by modulating intestinal microbiota and SCFA.	↑*Bacteroides fragilis* group ↑*Clostridium leptum* subgroup (Clostridium cluster IV) ↑*Clostridium coccoides* group (*Clostridium* cluster XIVa)	Prevents colitis caused by TNBS	(171)
**Prebiotic**							
Chitooligosaccharides + *Clostridium butyricum*	DSS-induced acute UC	16S rDNA	Stool	Exhibit significant anti-inflammatory and antioxidant properties, increase the expression of tight junction proteins, block the TRL-4/NF-κB/MAPK signaling pathway, and alter the amount and composition of gut microbiota	↑*Muribaculaceae* ↑*Lactobacillus* ↑*Clostridia_UCG-014*, *Turicibacter* ↑*Lachnospiraceae_* *NK4A136* ↑*Butyricicoccus*	Ameliorate colitis	(170)
Fructooligosaccharides	Colitis in HLA-B27 transgenic rats	16S rRNA, 16S rDNA	Caecum, stool	FOS altered the gut microbiota, leading to reduced chronic intestinal inflammation	↓Enterobacteriaceae ↑*Enterobacteriaceae* ↓*Clostridium difficile* ↑*Bifidobacterium* ↑*Bifidobacteria* ↓*Clostridium cluster IV*	Reduce colitis	(166, 167)
Chitooligosaccharides +*Bacillus coagulans*	DSS-induced colitis	16S rRNA high-throughput	An approved model for IBD	Alter cytokines, preserve mucin and tight junction protein expression, encourage intestinal barrier healing, control gut microbiota composition, and enhance SCFA synthesis.	↑*Ruminococcus* ↑*Akkermansia*	Attenuate DSS-induced UC	(263)
**β-glucans**							
YBNs@PDA	DSS-induced colitis	16S rRNA	Stool	Restores epithelial barriers, lowers ROS levels, and regulates gut microbiota	↑*Lachnospiraceae NK4A136* ↑*Bifidobacterium*	Mitigates DSS-induced colitis	(174)
**Resistant starch**							
PSPRS	DSS-induced colitis	16S rDNA high-throughput	Stool	Reduce pro-inflammatory cytokines, enhance SCFA and anti-inflammatory cytokine generation, and restore the disrupted gut flora	↑*Lactobacillus*, ↑*Alloprevotella*, ↑*Lachnospiraceae*_ NK4A136_group, ↑*Bifidobacterium*, ↑ ratio of *Firmicutes* to *Bacteroidetes*, ↓*Bacteroides*, ↓*Staphylococcus*, ↓*Akkermansia*	Treats colitis brought on by DSS	(176)
Dietary pectin	Salmonella typhimurium-induced colitis	16S rRNA	Caecal contents	Reduces H2O2 and MDA levels, decreases inflammatory cytokines, increases the abundance of intestinal tight junction proteins and CoQ10b expression, and inhibits TLR2-NF-κB signaling.	↓*Salmonella*	Ameliorate colitis caused by Salmonella typhimurium.	(178)
**Insoluble**							
**Cellulose**							
High-cellulose	DSS-induced colitis	16s rDNA	Stool	Increases mucus synthesis by goblet cells and has prebiotic effects on *A. muciniphila*	↑*Akkermansia*	Prevents DSS-induced colitis	(13)
Cellulose	DSS-induced colitis	16s rDNA	Stool	Controls the gut microbiota, lowers TNF-α and NF-κB expression, and raises PPAR-γ and IL-10 expression	↑*Bacteroides* ↑*norank_f__Muribaculaceae* ↑*Lactococcus* ↓*Faecalibaculum* ↓*unclassified_f__Lachnospiraceae*	Reduces DSS-induced colitis	(179)
**Hemicellulose**							
Xylan butyrate ester	DSS-induced UC	16S rRNA	Stool	Controls gut microbiota, increases GPR109A protein expression, inhibits HDAC activity, and promotes anti-inflammatory activity by activating autophagy pathways and inhibiting NF-κB	↓*Oscillibacter* ↓*Ruminococcaceae UCG-009* ↓*Erysipelatoclostridium* ↓*Defluviitaleaceae UCG-01* genera	lessens intestinal inflammation and damage	(182)
**Lignin**							
Ferulic acid-derived lignin nanoparticle (FALNP)	DSS-induced colitis	16S rRNA	Stool	Modulates the intestinal milieu by scavenging ROS and modifying gut bacteria, increasing tight junction proteins and anti-inflammatory cytokines while lowering pro-inflammatory cytokines	↑*Lactobacillus* ↑*Lachnospiraceae_* *NK4A136_group*	Alleviates colitis	(181)

^&^Inulin has the potential to repair the gut barrier and reduce inflammation. DSS, dextran sodium sulfate; IBD, inflammatory bowel diseases; PHGG, partially hydrolyzed guar gum; PSPRS, resistant starch from purple sweet potato; UC, ulcerative colitis; YBNs@PDA, yeast β-glucan nanocomplex coated with bio-adhesive polydopamine.

### 5.2 Polyphenols

#### 5.2.1 Curcuminoids

Polyphenols, plant-based substances found naturally or as semi-synthetic or synthetic derivatives, have demonstrated positive health impacts and therapeutic uses in several chronic diseases (184). Healthy, sub-healthy, and sick people use over-the-counter natural products to treat and prevent chronic illnesses, with biomedical researchers and medicine developers focusing on dietary polyphenols like curcumin, a curry component, for treatment and prevention (185). The turmeric plant, Curcuma longa, produces curcumin, a polyphenol belonging to the ginger family. It has long been used in Ayurvedic treatments to treat a variety of illnesses, including anorexia, asthma, coughing, hepatic diseases, diabetes, heart ailments, wound healing, and Alzheimer’s (186). A recent study has shown that curcumin inhibits further body weight and colon length reduction in IBD mice while improving the disease activity index, colonic mucosal damage, and inflammatory infiltration. Curcumin changes the gut microbiota by raising *Akkermansia*, *Muribaculaceae_unclassified*, and *Muribaculum* levels and markedly increasing intestinal concentrations of propionate, butyrate, glycine, tryptophan, and betaine (15). Furthermore, curcumin’s amelioration of intestinal dysbiosis impacted hepatic metabolic performance and enhanced the pathways linked to butanoates, bile acids, glucagon, amino acids, and biotin metabolism (15). These modifications might make it easier to establish the gut-liver axis’s equilibrium. However, curcumin’s impact on the gut-liver axis remains unclear, necessitating further confirmation of genes and proteins linked to gut and hepatic metabolite changes (15). Moreover, the extensive metabolomics data may have overlooked some significant targets due to the limited screening of intrahepatic metabolites with major intestinal genera (15). In another study, curcumin therapy dramatically reduced tumor growth in AOM/DSS-induced CRC model mice while restoring colon length and structural morphology. In the CRC model mice, curcumin decreased the number of harmful bacteria such as *Ileibacterium*, *Monoglobus*, and *Desulfovibrio* while increasing the number of good bacteria such as *Clostridia_UCG-014*, *Bifidobacterium*, and *Lactobacillus* (187).

#### 5.2.2 Phenolics

Polyphenols, like phenolic acids, have shown much promise in helping people with IBD by changing the gut microbiota. For instance, a study revealed that dietary caffeic acid (CA) prevents the rise in the *Firmicute* to *Bacteroidetes* ratio and promotes *Akkermansia* in mice with DSS colitis (188). Nevertheless, the authors did not prove a direct link between the improvement in colitis and the rise in the fraction of Akkermansia populations (188). Therefore, a causal association must be studied in the future. Additionally, CA can significantly decrease the release of IL-6, TNFα, and IFNγ, as well as the colonic infiltration of CD3^+^T cells, CD177^+^ neutrophils, and F4/80^+^ macrophages by blocking the signaling of NF-κB (188). Another study also found that CA supplementation altered the gut microbiome composition, increasing the abundance of *Akkermansia, Alistipes*, and *Dubosiella* while decreasing *Turicibacter* and *Bacteroides*’ abundance (189). Additionally, the study discovered that while *Dubosiella* abundance rose after CA injection, the mechanism is not specified (189). Thus, additional research is required to investigate the mechanism of *Dubosiella* and whether it could impact colitis (189). In a different study, caffeic acid phenethyl ester (CAPE) has shown potential in reducing nonalcoholic fatty liver disease in obese mice by manipulating gut flora. Treatment with CAPE primarily boosted the genera *Helicobacter*, *Bilophila*, *Enterococcus*, and *Bacteroides* (190). CAPE partially alleviates obesity-related steatosis by inhibiting bacterial bile salt hydrolase activity through the gut microbiota-bile acid-FXR axis (190).

Gallic acid (GA) is another phenolic acid shown to alleviate colitis and improve gut microbial dysbiosis. Treatment with GA restored the number of *Bacteroidales*, *Enterobacterales*, and *Clostridiales.* High-dose GA treatments inhibited the activation of NF-κB and MAPK signaling pathways, which were seen in mRNA levels following DSS treatment (191). In an alternative study, GA alleviates synovial inflammation and fibrosis in knee osteoarthritis by affecting the populations of *Bacteroidia* and *Muribaculaceae*, as well as through the metabolic pathways related to arginine biology, glycerophospholipid metabolism, and sphingolipid metabolism (192). However, concurrent use of cytochrome P450 family two subfamily D member 6 substrate medications and supplements containing gallic acid may result in harmful herbal-drug interactions (193). Additionally, gallic acid compounds are also known to have negative effects, including cytotoxicity and mutagenicity (194).

#### 5.2.3 Catechin

Green tea is rich in catechins and polyphenol flavonoids, with the most effective catechin being epigallocatechin 3-gallate (EGCG) (195). Oral EGCG, a key bioactive ingredient in green tea, strengthens the intestinal barrier and reduces inflammation in mice with DSS-induced murine colitis. EGCG alters the gut microbiota by boosting the quantity of *Akkermansia* and butyrate production (196). In another study, diets rich in green tea polyphenols (GTP) worsened intestinal inflammation and carcinogenesis brought on by DSS. Furthermore, colitis mice fed 1% GTP showed signs of nephrotoxicity, as evidenced by a substantial increase in serum creatinine levels (197). Additionally, green tea catechins have been suggested to decrease intestinal medication absorption by blocking OATP uptake, increasing P-gp export activity, or reducing drug solubility (198). These imply that catechins may have a dual role in disease management.

#### 5.2.4 Stilbenes

Stilbenes, classified as a type of polyphenol, have demonstrated the ability to prevent IBD via modulating gut microbiota. One of the active ingredients in the Chinese medicinal herb *Polygonum multiflorum* Thunb is tetrahydroxystilbene-2-O-β-D-glucoside (THSG) (199). THSG treatments elicited a beneficial pharmacological response in mice with DSS-induced acute colitis by reinstating epithelial barrier integrity and diminishing the synthesis of pro-inflammatory cytokines. THSG treatments significantly increased Firmicutes and Bacteroidetes abundances, restoring gut microbiota composition disrupted by DSS by increasing the genus Lachnospiraceae (NK4A136) and decreasing the genera Helicobacter, Bacteroides, and Parabacteroides (200). Nonetheless, this study did not investigate the gut metabolites; thus, future studies are needed to explore the metabolites and their association with the gut microbiota.

Resveratrol (RSV), a stilbene, has shown promise in mitigating IBD through its interaction with gut flora, thereby alleviating DSS-induced IBD symptoms. In mice, RSV reduces metabolite dysregulation, improves microbiota variety and composition, increases tight junction molecules, and alleviates colitis’s clinical symptoms. Additionally, RSV reversed the DSS group’s decrease in *Bacteroidetes* and *Proteobacteria* and rise in *Firmicutes* (201). Although the authors concentrated on RSV as a possible treatment for colitis, other substances with similar qualities might provide comparable effects. Consequently, this creates a gap that needs to be filled in future research (201). Co-administration of RSV with the probiotic strain *Ligilactobacillus salivarius* Li01 (RSV+Li01) has also shown a positive anti-inflammatory impact in DSS-induced colitis mice, promoting the healing of different inflammatory lesions and gut microbiota composition. Mice fed RSV+Li01 had larger relative abundances of *Bifidobacterium*, *Akkermansia*, and *Muribaculum*, but *Helicobacter* spp. were reduced (202). In another study, RSV therapy inhibited CRC growth in azoxymethane and DSS mice, boosting anti-inflammatory CD4^+^ FOXP3^+^ (Tregs) and CD4^+^ IL10^+^ cells, decreasing proinflammatory Th1 and Th17 cell growth, and modifying the gut flora (203). Other studies have demonstrated that RSV and its derivatives can reduce liver fibrosis caused by inorganic mercury and alleviate hypertension generated by a high-fat diet by modulating the gut microbiota (204, 205). RSV has been shown to inhibit carcinogenesis; nonetheless, due to the inhibition or activation of specific cytochrome P450s, pharmacological quantities of RSV may exacerbate adverse drug responses or change the efficacy of medications (206). Furthermore, RSV (5 mg/kg) has been shown to extend platelet plug development in mice (207), implying there is a tendency to bleed when using it. RSV may prevent IBD by modifying the gut microbiota, but it can potentially have negative consequences; therefore, caution should be exercised while utilizing specific amounts.

#### 5.2.5 Proanthocyanidins

Grape seed extract, which primarily consists of polymeric and oligomeric proanthocyanidins, epicatechin and monomeric catechin, and gallic acid, is rich in proanthocyanidins (208). Interestingly, research has found that grape seed proanthocyanidin (GSP), a plant-derived polyphenol, enhances inflammatory indices and decreases intestinal permeability, consequently reducing chronic inflammation in dogs. GSP therapy enhanced the number of bacteria, such as *Ruminococcaceae*, *Faecalibacterium*, *Ruminococcus_torques_group*, and *Lachnospiraceae_NK4A136_group*, that can reduce inflammation and stimulate bile acid metabolism (12). Unfortunately, this study did not evaluate the activities of 7α-dehydroxylase or bile salt hydrolase. The exact mechanism by which GSP regulates bile acids through the gut flora is still unknown (12). Future research may be required to investigate the role of GSP in controlling bile acids through the gut flora in colitis mitigation.

#### 5.2.6 Anthocyanins

It has been demonstrated that anthocyanins, such as pelargonidin-3-galactoside (Pg3gal), which are derived from purple sweet potatoes, considerably reduce DSS-induced UC in mice by preventing intestinal epithelial cell pyroptosis and preserving the structural integrity of the gut microbiota. Pg3gal changed the gut microbiota’s dysbiosis caused by DSS by increasing *Firmicutes*, *Bacteroidetes*, and *Verrucomicrobia* and decreasing *Proteobacteria* and *Deferribacteria* (209).

#### 5.2.7 Tannins

Kitabatake et al. (210) discovered that persimmon-derived tannin reduces colon inflammation in UC by changing the immune system and microbiota makeup. Supplementing with tannins considerably enhanced the relative abundance of *Bacteroides* while decreasing that of *Enterobacteriaceae* and *Enterococcus*. Also, mice with colitis fed a tannin diet had higher levels of *Bifidobacteria*. Thus, future research should evaluate the function of *Bifidobacterium* since it is unclear how the increased bacteria in mice given tannin supplements contribute to the improvement of colitis (210). Furthermore, more research on how tannin supplementation increases *Bifidobacterium* during colitis is anticipated to shed light on how probiotics preserve gut homeostasis (210). Additionally, Liu and the team discovered that punicalagin, administered orally to mice, alleviated DSS-induced colitis and elevated *Lachnospiraceae_NK4A136_group* and *Bifidobacterium abundance* (211). Punicalagin significantly increased the level of D-ribose. *In vitro* experiments showed that D-ribose has anti-inflammatory and antioxidant properties (211).

#### 5.2.8 Flavonols

Flavonols such as quercetin have also shown potential to ameliorate IBD. Due to quercetin’s capacity to inhibit pro-inflammatory cytokines and alter gut microbiota, dietary quercetin supplementation has therapeutic effects on colitis caused by *Citrobacter rodentium*. In colon tissues, quercetin increased the synthesis of IL-10 while inhibiting the production of pro-inflammatory cytokines like IL-17, TNF-α, and IL-6. Quercetin administration greatly decreased the populations of *Fusobacterium* and *Enterococcus* while increasing those of *Bacteroides*, *Bifidobacterium*, *Lactobacillus*, and *Clostridia* (212). One of the study’s noted limitations is the lack of human studies. Therefore, Lin et al. (212) stated that future research involving human participants is needed to validate the effects of quercetin on inflammatory markers and provide a more comprehensive understanding of the changes in human gut flora caused by quercetin. Interestingly, quercetin has been coated with nanoparticles (NPs) for better results. Compared to quercetin, quercetin NPs are better at controlling gut microbiota and SCFAs to help mice with colitis caused by DSS. Quercetin NPs enhance mucus protein and goblet cell density, reduce colon inflammatory infiltration, improve TNF-α, IL-1β, and IL-6, raise IL-10 levels, decrease MPO levels, and restore intestinal barrier integrity. Butyric acid, propanoic acid, and acetic acid concentrations were all raised by quercetin NPs (213). Treatment with quercetin NPs lowered the amounts of *Proteobacteria* in mice with colitis caused by DSS while increasing the amounts of *Verrucomicrobia* (213). However, the low pH of the colon may adversely affect the bioavailability of quercetin NPs in patients with UC, especially during the active phase. Therefore, patients in remission or with minor disease should be given more consideration for quercetin delivery methods, and a combination of medications should be used to treat colonic pH imbalance (213). In a different study, researchers have found quercetin to have a hypoglycemic effect, reduce insulin resistance, alter the metabolites of db/db mice, repair the intestinal barrier, and rebuild the intestinal microbiota. Quercetin reduced the number of *Escherichia coli*, *Bacteroides*, Proteobacteria, and *Escherichia-Shigella* (214). Conversely, taking quercetin together with warfarin has been shown to increase blood-thinning effects (215), implying there is a tendency for bleeding when used together.

**FIGURE 5:**
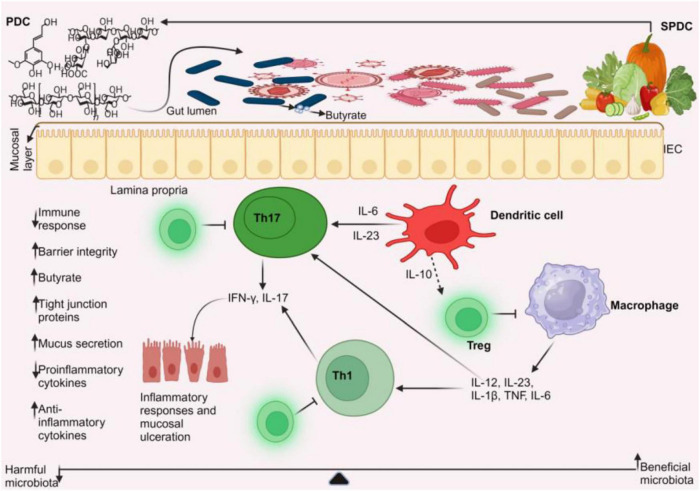
Plant-based management of IBD. Plant-based dietary compounds can help treat IBD by altering the gut microbiota. The beneficial microbiota stimulates dendritic cells to release anti-inflammatory cytokines, such as interleukin-10 (IL-10). This release of IL-10, in turn, promotes the development of regulatory T cells. These regulatory T cells prevent the activation of Th1, Th17 cells, and macrophages from producing pro-inflammatory cytokines, thereby helping to control IBD. This leads to decreased immune response, increased butyrate production, improved barrier integrity, and mucus secretion. PDC, plant-based dietary compounds; SPDC, sources of PDC.

Another flavonol that has been shown to reduce IBD is myricetin. Yang and colleagues discovered that myricetin controlled the gut microbiota composition in prediabetic mice, preventing DSS-induced colitis. The relative abundance of Bacteroidetes increased while the relative abundance of Proteobacteria declined dramatically after myricetin treatment (216). Furthermore, myricetin therapy elevated SCFAs like acetic, propionic, and butyric acids (216). Although altering the gut microbiota may positively impact the prevention of colitis, this investigation lacked clinical proof. Thus, Yang et al. (216) have proposed future clinical trials to determine the safety and efficacy of myricetin therapy over a lengthy duration. In a different investigation, myricetin modulated the gut-liver axis to have an anti-atherosclerotic effect (217). Myricetin decreased the abundance of genera linked to obesity, such as *Rikenellaceae_RC9*_*gut_group* and *Alistipes*, while increasing that of probiotics *g_Lachnospiraceae_NK4A136* (217). Table 3 summarizes the role of polyphenols in regulating the gut microbiota to prevent IBD. Overall, studies have shown that plant-based dietary components reduce experimental colitis by altering the gut microbiome, increasing beneficial bacteria, and decreasing harmful ones. This process maintains the homeostatic equilibrium of gut microorganisms (Figure 5).

**TABLE 3:** Polyphenols that impact the gut microbiota modulation in colitis.

Polyphenols	Model	Sequencing method	Sample	Mechanism of action	Microbiota signatures	Outcome	References
**Curcuminoids**							
Curcumin	DSS-induced acute colitis	16S rDNA	Stool	Enhances SCFAs, amino acids, glycolysis/gluconeogenesis, and amino acid metabolism pathways, while improving intestinal dysbiosis and liver metabolic diseases	↑*Akkermansia* ↑*Muribaculaceae_unclassified* ↑*Muribaculum* levels	Improves hepatic metabolism issues and intestinal dysbiosis	(15)
**Phenolic acids**							
Caffeic acid	DSS-induced colitis	16S rRNA	Stool	Reduces immune cell infiltration and inflammatory cytokine release while inhibiting NF-κB signaling pathways	↑*Akkermansia* ↓*Firmicute* to *Bacteroidetes* ratio	Ameliorates DSS-induced colitis	(188)
Caffeic acid	DSS-induced colitis	16S rRNA	Colonic digesta	Reduces pro-inflammatory cytokines, increases anti-inflammatory cytokines and antioxidants, and activates the Nrf-2/HO-1 pathway.	*↓Bacteroides* ↓*Turicibacter* ↑*Alistipes* ↑*Dubosiella* ↑*Akkermansia*	Ameliorates DSS-induced colitis	(189)
Gallic acid	DSS-induced colitis	16s rRNA	Stool	Increases anti-inflammatory cytokines, reduces pro-inflammatory cytokines, and inhibits the NF-κB and MAPK signaling pathways	↑*Bacteroidales ↑Enterobacterales* ↑*Clostridiales*	Alleviates colitis	(191)
**Catechin**							
epigallocatechin 3-gallate	DSS-induced colitis	16S rRNA	Stool	Reduce inflammation in the colon in a gut microbiota-dependent way	↑*Akkermansia*	Reduces in colitis	(196)
**Stilbenes**							
2,3,5,4’-tetrahydroxystilbene-2-O-β-D-glucoside	DSS-induced acute colitis	16S rDNA	Stool	Reduces pro-inflammatory cytokines, boosts anti-inflammatory cytokines, enhances tight junction proteins, and controls gut flora	↑Firmicutes ↑Bacteroidetes ↑Lachnospiraceae ↓Helicobacter ↓Bacteroides ↓Parabacteroides	Suppresses acute colitis caused by DSS	(200)
Resveratrol	Azoxymethane and DSS-induced colitis CRC	16S rRNA	Colonic flush contents	Modifies the microbiome to promote butyrate synthesis, decreases histone deacetylases and the inflammatory T cell response, and increases Treg	↑*Ruminococcus* ↑*Akkermansia* ↑*Dehalobacterium* ↑*Anerostipes* ↑*Anaeroplasm* ↑*Blautia* ↑*Clostridium*	Attenuates inflammation-driven CRC	(203)
Resveratrol	DSS-induced colitis	16S rDNA sequencing	Stool	Modulates the microbiota-macrophage-arginine metabolism pathway	↑*Bacteroidetes ↑Proteobacteria ↓Firmicutes*	Mitigates DSS-induced IBD	(201)
Resveratrol + *Ligilactobacillus salivarius* Li01	DDS-induced colitis	16S rRNA	Colon contents	Activate the AHR and tryptophan metabolism axis to boost anti-inflammatory impact.	↑*Bifidobacterium ↑Muribaculum ↑Akkermansia* *↓Helicobacte*r spp	Attenuates colitis	(202)
**Proanthocyanidins**							
Grape seed proanthocyanidin	Intestinal inflammation in canines	16S rRNA	Stool	Changes gut microbial composition and enhances bile acid metabolism	↑*Ruminococcaceae* ↑*Faecalibacterium* ↑*Ruminococcus_torques_group* ↑*Lachnospiraceae_* *NK4A136_group*	Reduces intestinal inflammation	(12)
**Anthocyanins**							
Pelargonidin-3-galactoside	DSS-induced colitis	16S rRNA	Stool	Reduces pro-inflammatory cytokines and pyroptosis while improving gut microbiota structural integrity	↑*Firmicutes* ↑*Bacteroidetes* ↑*Verrucomicrobia* ↓*Proteobacteria* ↓*Deferribacteria*	Relieves colitis brought on by DSS	(209)
**Flavonols**							
Quercetin	*Citrobacter rodentium*-induced colitis	16S rRNA	Colonic contents	Increase anti-inflammatory cytokines, reduce pro-inflammatory cytokines, and/or alter the gut microbiome	↑*Bacteroides* ↑*Bifidobacterium* ↑*Lactobacillus* ↑*Clostridia* ↓*Fusobacterium* ↓*Enterococcus*	Controls *Citrobacter rodentium*-induced inflammation	(212)
Quercetin NPs	DSS-induced colitis	16S rRNA	Colonic contents	Reduces inflammation, improves gut microbiota, and repairs the intestinal barrier by targeting the colon	↑*Verrucomicrobia* ↓*Proteobacteria*	Alleviates colitis caused by DSS	(213)
Myricetin	DSS-induced colitis in prediabetic mice	16S rRNA	Stool	Suppresses proinflammatory cytokines, increases the expression of tight junction proteins, modulates the gut flora, and increases SCFA synthesis	↓Proteobacteria, ↑*Bacteroidetes.*	Reduces inflammation caused by DSS	(216)
**Tannins**							
Persimmon-derived tannin	DSS-induced colitis	16S rRNA	Stool	Inhibit the inflammatory reaction and modify the microbiome	↑*Bacteroides* ↓*Enterobacteriaceae* ↓*Enterococcus*	Reduces intestinal inflammation in UC	(210)
Punicalagin	DSS-induced colitis	16S rRNA	Stool	Regulates gut flora and metabolites (D-ribose) to relieve colitis	↑*Lachnospiraceae_* *NK4A136_group* ↑*Bifidobacterium*	Relieves colitis	(211)

DSS, dextran sodium sulfate; LPS, lipopolysaccharide; NPs, nanoparticles.

## 6 Clinical evidence of plant-based dietary compounds

For patients with quiescent UC, curcumin appears to be a safe and promising drug for sustaining remission. Curcumin reduced the morbidity associated with UC by enhancing the endoscopic and clinical activity indices. For 6 months of treatment, 2 out of 43 patients who took curcumin experienced a relapse, while 8 out of 39 patients in the placebo group did the same (218). Another clinical trial found that curcumin supplementation in mesalamine medication was more effective than a placebo combination in causing clinical and endoscopic remission in mild-to-moderate active UC patients. There were no noticeable negative effects (219). This shows that combination therapy with plant-based dietary compounds and IBD medications is safe and effective. A recent study found a novel curcumin derivative called Theracurmin^®^, with a 27-fold higher absorption rate than natural curcumin powder, clinical and endoscopic effectiveness, and a positive safety profile in patients with active CD (220). A different study also showed that curcumin, a safe and effective adjuvant drug, lowers anti-ds DNA and IL-6 levels, thereby reducing inflammation and autoimmune activity in people with systemic lupus erythematosus (221). Nevertheless, some prebiotics used in a clinical setting showed no improvements. Benjamin et al. (222) found that fructooligosaccharides, despite affecting dendritic cell function, did not have a therapeutic effect in patients with active CD (223).

Despite the limited clinical studies on the role of plant-based dietary compounds in mitigating IBD, other clinical trials and investigations have demonstrated the potential for plant-based diets to prevent various disorders, including IBD. Additionally, researchers have integrated plant-based diets with other drugs to achieve optimal results. Chiba et al. (224) discovered that patients undergoing infliximab medication combined with a lacto-ovo-semivegetarian diet exhibit a reduced mean CD disease activity index score at week 6 post-admission. The average level of C-reactive protein upon admission dropped. Of the cases, 46% (19/41) had mucosal healing. A similar study found that for over half of CD patients, infliximab with a plant-based diet as first-line treatment produced an unparalleled relapse-free course. Three regular infliximab infusions combined with a plant-based diet produced remission in 24 consecutive newly diagnosed adult patients with CD while they were in the hospital (225). Another study also found that relapse rates in UC significantly decreased following lacto-ovo-semivegetarian diet induction therapy compared to standard therapeutics. The relapse rate for initial episode cases of UC after therapy with a lacto-ovo-semivegetarian diet was lower than traditional treatment, with rates of 14% at 1 year and 27% at 5 years. None of the patients reported any significant side effects that a lacto-ovo-semivegetarian diet might have caused (226). Also, 77% of patients with mild or remission of UC who were given nutritional advice and placed on a plant-based diet during a 2-week hospital stay reported improvement, including bloody stool elimination or reduction. At 1, 2, 3, 4, and 5 years of follow-up, the cumulative recurrence rates were 2, 4, 7, 19, and 19%, respectively. None of the patients adhering to plant-based diets encountered any harmful effects (227). These suggest that plant-based diets could be beneficial for use in clinical settings.

## 7 Role of personalized nutrition in IBD

Nutrition plays a crucial role in the development and progression of disease, making it a potential therapeutic approach to suppress inflammation and symptoms. Given that IBD is a diverse condition clinically and molecularly, tailoring dietary recommendations may be essential to bringing about long-lasting dietary behavior changes that enhance nutritional status and address gut inflammation and abdominal symptoms on a personal basis (228). With the help of data on individual traits like age, insulin sensitivity, or gut flora, personalized nutrition creates individualized dietary recommendations to help patients make positive, long-lasting diet changes (229).

### 7.1 Individualized triggers

It is crucial to remember that some foods might cause IBD to worsen or subside. For instance, UC risk was linked to a diet imbalance that included low vegetable and high sugar and soft drink intake (230). Opstelten and the team also found that although a distinct dose-response association was not demonstrated, milk consumption may be linked to a lower risk of getting CD (231). Recently, there has been insufficient proof to conclude that dairy products and milk affect the occurrence and progression of IBD (232). Increased consumption of highly processed foods was strongly correlated with an increased incidence of IBD (233, 234). In the case of plant-based diets, some plant-based dietary compounds have been demonstrated to worsen and exacerbate IBD and cause carcinogenesis (164, 172, 197). Therefore, this will help clinicians/individuals to plan their nutrition to prevent IBD exacerbation.

### 7.2 Nutrient deficiencies and quality of life

Malnutrition is believed to affect 20–85% of people with IBD. Among the many causes of malnutrition in IBD patients are decreased oral meal intake, intestinal bacterial overgrowth, chronic blood and protein loss, and malabsorption. Clinical outcomes, response to treatment, and quality of life are all negatively correlated with poor nutritional status, selective malnutrition, and sarcopenia. Radiological evaluation, functional capacity measurement, and dietetic evaluation involving daily caloric intake and energy expenditure should all be part of the nutritional assessment (235). This reduces IBD exacerbations and improves the quality of life (QoL) for IBD patients. Along with malnutrition brought on by the inflammatory nature of the illness, a significant number of IBD patients exhibit restricted dietary practices (41–93%) and food avoidance (28–89%), which have a negative influence on their QoL when it comes to eating (236, 237). This may lead to nutritional deficiencies, as reported in studies (238, 239), where patients with IBD (active or in clinical remission) developed nutrient constraints including vitamin C, copper, niacin, and zinc, among others. Similarly, individuals who consume fewer or no animal products may be susceptible to dietary deficiencies in protein, calcium, iron, iodine, zinc, vitamin B12, vitamin D, and omega-3 fatty acids. This deficit can result in immediate and long-term health issues (240). Research conducted in Switzerland, Spain, and Germany identified deficiencies in vitamins and minerals, including vitamins B6, B12, and niacin, among plant-based groups (241–243). Therefore, inadequate consumption of vital nutrients highlights the necessity for more effective public health initiatives and enhanced nutrition education, irrespective of eating habits (59).

## 8 Limitations of plant-based dietary treatments

Although fibers offer many health benefits, not all are equal, and some patients with IBD report intolerance to specific types of fiber (244). Armstrong et al. (244) found that in a subgroup of IBD intestinal biopsies cultivated *ex vivo*, unfermented dietary β-fructan fibers produced proinflammatory cytokines and immune cells. The NLRP3 and TLR2 pathways had to be activated to release the proinflammatory response to intact β-fructan. Patients without fermentative microbe activity experienced negative effects. Other studies (164, 172, 197, 245) have also shown the negative impacts of plant-based diets on IBD by way of exacerbating the condition and causing carcinogenesis. This may lead to the avoidance of such dietary compounds. Another study (246) demonstrated that plant dietary compound combinations may affect the efficacy of others in reducing colitis. Additionally, studies (218, 224, 225, 227) used smaller sample sizes to determine the effectiveness of plant-based diets in IBD. These studies have indicated the necessity for greater sample sizes in subsequent research.

Regarding the preclinical studies, this review revealed several shortcomings. One significant limitation, for example, was the absence of clinical studies or trials on the plant-based dietary components (except for curcumin). Additional limitations included the use of a single dose in gut microbiome studies, the non-selectivity of nanoparticles in inflammatory areas, the exclusion of important microbiota from studies, and the incomplete and ambiguous mechanisms underlying the effects of some dietary components on metabolites and the gut microbiota. There were no studies on plant-based chemicals and intestinal fungus or virome.

## 9 Future directions

Due to nutrient deficiencies that may arise from plant-based dietary compound consumption, it is necessary to design a plan to help prevent such issues. Therefore, in this situation, the ability to create a customized meal plan for each patient will likely improve disease treatment, boost adherence since patients are more receptive to individualized approaches, and be more flexible (228). More clinical trials on nanoparticles in IBD patients should be explored. However, when exploring plant-based dietary components as nanoparticles for potential clinical applications, IBD patients’ pH should be considered, as these compounds act best at high pH (preclinical studies), whereas IBD patients typically have low pH (247). As a result, combining these nanoparticles with another treatment regimen to aid in colonic pH regulation may enhance the nanoparticles’ therapeutic effects. Additionally, more intestinal microbiota should be used to evaluate important microbiota. Future studies should include more human studies with larger sample sizes to confirm the role of these dietary compounds in IBD. Most studies centered on gut bacteria; thus, more research is needed on how plant dietary compounds regulate gut fungi and virome. Uncertain and incomplete mechanisms involving the research of plant-based components on the gut microbiota should be elucidated, and more dosages, rather than a single dose, should be investigated in gut microbiome studies.

## 10 Conclusion

Plant-based dietary components have been demonstrated to reduce IBD symptoms by increasing anti-inflammatory cytokines, decreasing pro-inflammatory cytokines, lowering oxidative stress, and improving barrier function. These compounds prevent IBD by activating/inhibiting multiple signaling pathways, including TGF-β/Smad, TRL-4/NF-κB/MAPK, TLR2-NF-κB, autophagy, pyroptosis, glycolysis/gluconeogenesis and amino acid metabolism, Nrf-2/HO-1, microbiota-macrophage-arginine metabolism, and bile acid metabolism. Furthermore, these dietary components aid in the formation of SCFAs, which promote the development of Tregs, thereby alleviating IBD. While many plant-based nutritional components have been demonstrated to reduce the severity of IBD, others have been shown to increase it or cause cancer. This will aid clinicians when planning diets for their patients. However, the favorable effects make plant-based dietary components a promising alternative for IBD treatment in clinical settings. Increased beneficial microbiota has also been linked to butyrate and anti-inflammatory marker production, whereas bad microbiota leads to inflammatory marker production. Emerging evidence has shown the promising role of dietary compounds used as nanoparticles or dietary compounds encapsulated in nanoparticles for effective treatment of IBD. These nanoparticles are safe and non-toxic in preclinical studies, warranting further studies in clinical settings for IBD patients.
